# Association of *COMT* Val158Met Polymorphism with Fibromyalgia in Khartoum State, Sudan

**DOI:** 10.1155/2023/7313578

**Published:** 2023-06-03

**Authors:** Safaa Mamoun Abdelmageid, Faisal Mousa Alamir, Hassan Yousif Abdelrahman, Hind Mohamed Abushama

**Affiliations:** ^1^Department of Zoology, Faculty of Science, University of Khartoum, Khartoum, Sudan; ^2^Royal Care Hospital, Khartoum, Sudan

## Abstract

Fibromyalgia (FM) is a disorder characterized by chronic musculoskeletal pain, fatigue, and cognitive problems. Neurotransmitters, mainly catecholamines, appear to be involved in regulating the etiology of FM. Catechol-O-methyltransferase (*COMT*) is involved in catabolizing catecholamines such as norepinephrine. The most common variant studied in the *COMT* gene is the valine (Val) to methionine (Met) substitution at codon 158. This is the first study in Sudan addressing FM cases and genetic susceptibility to the disease. We aimed in this study to investigate the frequency of *COMT* Val 158 Met polymorphism among patients with FM, rheumatoid arthritis, and in healthy individuals. Genomic DNA from forty female volunteers was analyzed: twenty were from primary and secondary FM patients, ten were from rheumatoid arthritis patients, and ten were from healthy control. FM patients' age was ranging from 25 years to 55 with a mean of 41.14 ± 8.90. The mean age of the rheumatoid arthritis patients and healthy individuals was 31.3 ± 7.5 and 38.6 ± 11.2, respectively. Samples were genotyped for *COMT* single nucleotide polymorphism rs4680 (Val158Met), using the amplification-refractory mutation system (ARMS-PCR). Genotyping data have been analyzed using the Chi-square and Fisher exact test. The most common genotype among the study participants was the heterozygous Val/Met found in all participants. It was the only genotype found in the healthy participants. The genotype Met/Met was found only in FM patients. The genotype Val/Val was found only in rheumatoid patients. Analyses have shown no association between the Met/Met genotype and FM, and this could be due to a small sample size. In a larger sample size, a significant association could be found as this genotype was shown only by FM patients. Moreover, the Val/Val genotype, which is shown only among rheumatoid patients, might protect them from developing FM symptoms.

## 1. Introduction

FM is an idiopathic medical condition characterized by a low pain threshold and chronic disabling pain fatigue in the joints and tenderness of muscles that persist for more than three months [1]. FM is not just a discrete disorder but rather a wide spectrum of disorders that are controlled by environmental factors [2]. Most patients are diagnosed with FM after experiencing psychological or physical trauma. One of the physical traumas that trigger FM is the physical pain caused by rheumatoid arthritis and other autoimmune diseases [3]. Musculoskeletal pain is considered the main symptom of primary FM. If additional disorders appear beside the musculoskeletal pain, then the condition is considered secondary FM that appears with another primary disease usually with rheumatoid arthritis [4, 5]. A general observation of familial aggregation among FM patients has been noticed, which might indicate the existence of the genetic etiology of the disease [6]. It has been studied that the variations in specific neurotransmitter-related genes increase susceptibility to FM via hypersensitivity to the peripheral painful stimulus by the central nervous system (CNS). This is further demonstrated by genome-wide analysis of single nucleotide polymorphisms and copy number variants in FM patients [7].

COMT is one of the most studied genes in FM. This gene is involved in breaking down catecholamine neurotransmitters and therefore, in modulating pain perception by the CNS [8]. Accordingly, patients have elevated levels of catecholamines and higher levels of basal norepinephrine [9]. Some single nucleotide polymorphisms (SNPs) in the *COMT* gene give rise to defective enzymes that have been suggested to contribute to FM susceptibility and symptom severity. The nonsynonymous variant in the *COMT* gene Val to Met substitution at codon 158 is the most studied one. The presence of the Met variant leads to a low enzyme activity, which is fourfold reduced than the normal activity, due to the increased thermal ability of the physiological temperature. This in turn increases the norepinephrine levels in the prefrontal cortex [10].

A satisfactory number of studies have identified associations between the low-activity Met158 allele and several pain phenotypes [11–15]. There are three genotypes of the *COMT* gene Val158Met enzyme as illustrated in Table 1 [10]. The present study investigates the possible association between Val158Met and the risk of developing FM in Sudanese patients.

There is a considerable ethnic and cultural diversity within Sudan. Sudanese lie under three main categories (Arab, Afro-Arab, and African) [16]. Under these categories, there are five large tribes: Fur, Beja, Gaalin, Hawazma, and Messeryia. The Nile Valley has a long history of succession of different groups, coupled with demographic and migration events, which remain to be fully examined on a genetic level. These groups include people with an established history in the area (for example, Nuba and Nilotic) and groups that migrated to the area in relatively recent times (for example, Hausa, Copt, and Arab) [17].

### 1.1. Rational

This is the first study in Sudan addressing FM case appearance and genetic susceptibility to the disease. The first trigger to conduct this study was the appearance of the Sudanese FM association on social media. The Sudanese FM association has grabbed great attention. Its followers were around four hundred and fifty-five supporters at the time of the study (https://www.facebook.com/groups/198486480584263/?ref=share). The platform includes FM patients who would like to share their suffering and experiences about suitable treatments and pain relief. The disease is described as multiple disorders that fall under the FM umbrella. The disease symptoms vary a lot. Studying the genetic bases of FM might help in developing personalized medications for each category of patients who are genetically different. In Sudan, there are around five hundred different ethnic groups that vary widely compared to other populations [16]. The present study is a pilot study to investigate the association of *COMT* rs4680 Val158Met SNP with FM as it is the most studied SNP in previous studies and because there is no specific fund allocated for this study to look for any additional SNPs. Future studies can look at other polymorphisms in the *COMT* gene.

## 2. Materials and Methods

The Ethics Committee of the Sudanese Ministry of Health has approved the conduct of this study. The study participants were recruited mainly from Royal Care Hospital in Khartoum State. It is a private hospital that receives and diagnoses most of FM cases in the rheumatoid clinic. It is one of the only two clinics that diagnose FM. Conducting a research work in private hospitals is not allowed most of the time according to private hospitals' policy. Participants were approached from the Royal Care Hospital's rheumatoid clinic registry in Khartoum State. They have been oriented about the objectives of the study before signing the informed consent.

### 2.1. Inclusion Criteria

#### 2.1.1. FM Patients

Female participants were diagnosed by a professional rheumatologist in the hospital as FM patients following the 1990 American College of Rheumatology (ACR) criteria for diagnosing FM. The diagnosis is divided into two parts. First, the widespread pain was assessed by the widespread pain index (WPI) which relies on the patient's report of the pain felt in 19 different parts of the body during the past weeks. Each part has been assigned 1 point if the pain was felt on it. The maximum score given was 19/19. Secondly, the diagnosis was confirmed by the clinical examination of the tender points. The patient should have at least 11 out of the total 18 tender points [18].

#### 2.1.2. Rheumatoid Arthritis Patients

Female participants diagnosed with rheumatoid arthritis were included in this study. Their diagnosis has been conducted according to the American college of Rheumatology diagnosis guidelines for diagnosing rheumatoid arthritis 2010.

#### 2.1.3. Healthy Individuals

Healthy individuals are all females' copatients of FM participants who were proven to be healthy after conducting clinical and laboratory investigations.

### 2.2. Sample Collection

This is a pilot hospital-based study conducted from 2018 to 2019. There is no previous information on disease occurrence or prevalence. There are only two rheumatoid clinics that diagnose FM in the country; one of them is our study clinic. The samples and data have been collected from a private hospital and there are strict regulations about data sharing outside the hospital. Participants have been recruited from Royal Care hospital of Khartoum/Sudan. Data related to clinical symptoms were recorded by a specialized nurse in the hospital. We were able to enroll 40 participants who had been selected as not having any previous treatment including serotonin noradrenaline reuptake inhibitors (SNRIs) or antidepressant receipts. Out of the admitted cases at the rheumatoid clinic in the hospital, 20 participants have met the 1990 American college of rheumatology criteria for diagnosing FM. Ten of the copatients from the same socioeconomic background have been taken as controls and were diagnosed at the same clinic as not having FM. The study also included 10 patients clinically diagnosed with rheumatoid arthritis according to the American College of Rheumatology criteria for diagnosing rheumatoid arthritis (2010) who were recruited from the same hospital. Three ml of venous blood were collected from each participant before taking any type of treatment.

### 2.3. Genotyping

DNA were extracted from blood samples using the salting-out protocol as described elsewhere [19] Afterwards, we conducted a spectrophotometric quantification for the DNA samples using NanoDrop 2000c, ThermoFisher. All the samples were stored at −20°C.

A trial has been made up first to setup the genotyping and PCR reaction conditions using the phenotype-blind genotype process. We used a DNA concentration of 50 ng/*μ*L in the PCR reaction. The samples were genotyped for the *COMT* gene Val158Met SNP using the ARMS technique. It has been amplified using tetra primers as described by a previous study [20]. P1 forward: CCAACCCTGCACAGGCAAGAT, P2 reverse: CAAGGGTGACCTGGAACAGCG, P3 forward: CGGATGGTGGATTTCGCTGaCG, P4 reverse: TCAGGCATGCACACCTTGTCCTTtAT. Further validation of the ARMS technique has been made by conducting convention PCR using each pair of primers (forward and reverse) in two separate PCR reactions.

PCR reactions were carried out in a total volume of 25 *μ*l, containing 3 *μ*l of genomic DNA containing 3 *μ*l of genomic DNA (50 ng/*μ*l), 5 *μ*l of PCR premix (iNtRon® Biotechnology), 5 *μ*l of each primer (10 pmol), and 15 *μ*l nuclease-free water. Positive and negative controls were added to validate the results. PCR was carried out in an AERIS™ ESCO® PCR machine, with the following program: initial denaturation at 94°C for 4 min, then 30 cycles of 94°C for 30 sec, followed by 62°C for 30 sec and 72°C for 20 sec, and a final extension at 72°C for 5 min.

5 *μ*l of the PCR product and 1 *μ*l of a 100 bp DNA ladder were loaded on 1.5% agarose gel electrophoresis mixed with 1 mg/ml ethidium bromide. The gel was viewed under UV light. Using SPSS, we computed Pearson's *χ*^2^ and Fisher's exact test to analyze the differences in SNP genotype frequency between FM and controls. Significance was set at *p* < 0.05. The association between allele frequency and the disease status was checked using the SNPass package.

## 3. Results

In the present study, a total of 40 samples from Khartoum city from Royal Care Hospital were collected, 20 blood samples were collected from FM patients, 10 from rheumatoid arthritis patients, and 10 from healthy individuals. The age of the FM patients was ranging from 25 years to 55 with a mean of 41.14 ± 8.90 with a normal distribution (Shapiro–Wilk *p* value = 0.7031). Their weight was normal (60−70 kg) according to their age.  Table 2 illustrated the data collected from the study participants.

Rheumatoid arthritis patients and healthy individuals were all females with a mean age of 31.3 ± 7.5 and 38.6 ± 11.2, respectively. The participants were all Sudanese females from a middle socioeconomic class and were all of the same ethnic group. All study participants were nonsmokers and nonobese.

FM symptoms varied among participants but they all shared a chronic widespread pain, unrefreshing sleep, physical exhaustion, and impaired cognitive (memory) abilities and had at least a chronic widespread pain all over the body and a range of 11 to 18 tender points. Signs of depression have also been noticed.

**Figure 1:**
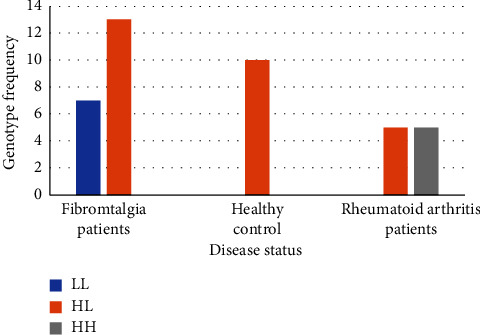
The frequencies of the three genotypes of *COMT* Val158Met, HH, HL, and LL among the study participants. HH represents the genotype of normal homozygous Val; HL represents the heterozygous; LL represents the mutant homozygous Met. The most common genotype among the three categories was the heterozygous genotype HL which represents 70% of the samples with heterozygosity = 0.5050633.

All collected samples were successfully genotyped. *COMT* gene Val158Met SNP genotyping has revealed three bands for heterozygote (626, 451, and 222 bp), two bands with homozygous Met (626, 222 bp), and two bands with homozygous Val (626, 451 bp). The most common genotype among all study participants was the heterozygous genotype HL which is represented by 70% of the samples with heterozygosity = 0.5050633 as illustrated in Figure 1. The Hardy–Weinberg equilibrium *p* value is 0.0015.

Among FM patients, 65% carried the heterozygous genotype HL, and 35% showed the homozygous genotype LL while none had shown the HH genotype. All the healthy study participants (100%) have shown the heterozygous genotype, whereas 50% of the rheumatoid patients showed the genotype HL, and the other 50% showed the homozygous HH. None of the rheumatoid patients have shown the LL genotype as illustrated in Table 3.

Fisher's exact test had shown a significant difference in the distribution of the *COMT* Val158Met genotypes among the three categories under study with a *p* value = 0.033 as shown in Table 3. The HH genotype has only been shown in rheumatoid patients.

When we combined the genotype frequency of FM and rheumatoid individuals and compared it with healthy individuals' genotype frequency, the difference was not significant using fisher's exact test (*p* value = 0.064). Allele frequency has been found to be 0.52 for the L allele and 0.48 for the H allele There was no significant association between the allele frequency and the FM using the SNPass package (log-additive model *p* value = 0.063) as well as using logistic regression (*p* value = 0.738).

## 4. Discussion

FM patients were diagnosed according to ACR criteria in 1991. Symptoms varied among participants but they all shared a chronic widespread pain, unrefreshing sleep, physical exhaustion, and impaired cognitive (memory) abilities. Most of the study participants had 18/18 tender points and the least one had 11/18. This variation in symptoms has also been observed among patients from other different populations in previous studies [2, 21]. The new diagnostic measures for FM include unrefreshed sleep and fatigue which have nearly both been given equal weight. Depression is included as a trivial symptom [5]. All study participants are from the same ethnic group *Galieen*. They also showed no signs of obesity, and they did not report any disturbance in their sleep patterns.

Number of investigators thought that it is unclear whether these combined symptoms of FM are simply the consequence of chronic pain or whether they occur exclusively as a critical component of this disorder. Individual variations are also noticeable among patients, although chronic widespread pain remains the most prominent feature of FM.

All participants in this study were females as shown in findings from previous studies [2]. There were many explanations for this bias towards one type of gender. In general, positive tender points are more frequently described by women than by men in general [22]. Moreover, the higher rate of positive tender points can be interpreted by a lower pain threshold which is observed in women than in men [23]. Women usually complain about their somatic and psychological symptoms more than men [24]. Physicians mostly viewed FM as a feminine disease and may misdiagnose men when presented with chronic widespread pain. Moreover, men might not wish to be diagnosed as a known female condition [25].

The mean age of FM patients was found to be 41.14 ± 8.90. This is in accordance with the previous studies where FM was found to be affecting women in their middle age [3]. This might not truly reflect the situation as symptoms of FM prevalence range only between 2% and 4% in the general population. In addition to that, women between 40 and 60 years of age are the majority of individuals who visit the clinics.

All participants were from the middle class. FM patients are mostly of higher and middle-class socioeconomic status as measured by the earning rate per year [26].

The most common genotype among all study participants was the heterozygous form, HL, which reduces an enzyme with moderate activity [27]. This high heterozygosity rate was observed in previous studies [28]. There is a significant deviation in the genotype frequency from Hardy–Weinberg equilibrium, and this is clearly due to the small sample size under investigation in this study.

There is no significant difference when we combined the genotype frequency of FM and rheumatoid individuals and compared it with healthy individuals' genotype frequency. This finding has shown previously in number of studies [29–32]. Other factors that might affect this lack of association could be further studied in the future. In a meta-analysis study [29], the authors have indicated a possible role of the ethnic factor in the genetics of FM.

The genotype *COMT* Val158Met LL was present only in FM patients in 35% of them. The homoygous LL yield a defective enzyme that has low activity in degrading catecholamines. It has been found that in heterozygous individuals, HL, who experienced a physical or psychological trauma or long-term stress, their catecholamines levels will be activated and raised above normal levels, and then the H allele help to maintain this activation for a relatively longer time [2, 21]. A larger sample size is needed to verify the finding of the present study as demonstrated in other populations: Spanish [33], Turkish [27, 28], and Brazilian populations [11] which showed a significant association between *COMT* Val158Met and FM.

The heterozygous genotype HL has been found in 50% of the rheumatoid patients in this study, and the homozygous genotype HH has been found in the remaining 50%. It has been shown that people with the genotype HH have a higher pain threshold and cope better with stress compared to the LL carriers [28, 34]. The pain caused by rheumatoid arthritis is expected to be a trigger to develop FM in rheumatoid arthritis patients. The second allele, H, in the 50% homozygous rheumatoid patients, seems to protect them from getting FM. The HH carriers have a higher pain threshold which means a good perception of pain induced by rheumatoid arthritis [3, 35]. This might explain why this genotype is common among rheumatoid arthritis patients. In addition, this genotype has not been noticed in both healthy individuals and FM patients. Several studies have identified associations between the low enzyme activity in LL genotype individuals and several pain phenotypes [11, 13–15, 33, 36]. However, the observed associations were found to be modest and inconsistent which suggest that the activity of *COMT* might be modulated by other SNPs [37].

Currently, there is no available FM study among the Sudanese population. Any information about the condition will be valuable.

The effect of the *COMT* gene genotypes on the daily dynamic physiological mechanisms through moderating the chronic pain process needs further investigation. FM is mostly a feminine disease. *COMT* gene transcription is thought to be affected by many factors including estradiol, a female sex hormone that is produced in varying amounts throughout the menstrual cycle [38]. Our samples included women in age associated with premenopausal and perimenopausal status. Daily fluctuations in estradiol levels may affect *COMT* interactions. Such factors, besides the small sample size, are making constrictions for further elucidation of the study. However, the disclosure of this limited information about FM in Sudan will have a good impact on the future investigation of the disease status in Sudan. Other factors including environmental ones besides genetics influence should also be investigated as they might affect the etiology of FM as shown in previous studies [21]. The present study is a pilot study of a small sample size. The performance of multivariate logistic regression to include the environmental factors investigated will not be of significant value. However, it is important to conduct this investigation to highlight the existence of FM in the Sudanese community. In the future studies, environmental factors and social determinants will be studied extensively in a large sample size to evaluate the role of the studied SNPs on disease risk.

This study will also open a new avenue for FM symptoms awareness in Sudan and possible remedies that could be used to lower suffering. Evidence-based interdisciplinary studies give strong advice for ceasing smoking, aerobic exercise, and cognitive-behavioral therapies.

### 4.1. Limitations

The main limitation of this study is the sample size. The larger sample size would illustrate whether there is a genetic association between the *COMT* Val/Met 158 and FM. Moreover, detailed information about variations in pain tender points and investigation of other suspected *COMT* polymorphisms would add more strength to the statistical analyses of the study. Other factors such as experimentally controlling estradiol levels across a menstrual cycle would be valuable to be investigated.

## Figures and Tables

**Table 1:** *COMT* rs4680 Val158Met polymorphism and enzyme activity.

*COMT* gene polymorphism	Enzyme polymorphism	Enzyme activity
HH	Val/Val	High (the H/H (Val-158-Val) genotype generates a fully effective enzyme)

LH	Met/Val	Medium (the H/L (AG; Met-158-Val) genotype generates an intermediate-activity enzyme)

LL	Met/Met	Low (the L/L (AA; Met-158-Met) genotype produces a defective enzyme, which impairs the clearance of catecholamines from the CNS)

**Table 2:** Data collected from study participants.

	FM	RH	Healthy controls
Age	41.14 ± 8.90	31.3 ± 7.5	38.6 ± 11.2
Mean			

Education	(i) 95% undergraduate	(i) 98% undergraduate	(i) 91% undergraduate
	(ii) 5% postgraduate	(ii) 2% postgraduate	(ii) 9% postgraduate

Ethnic group	Galieen	Galieen	Galieen

Income	Range 350–400 USD per year	Range 350–400 USD per year	Range 350–400 USD per year

Marriage	(i) 74% married	(i) 68% married	(i) 73 married
	(ii) 10% divorced	(ii) 2% divorced	(ii) 4% divorced
	(iii) 16% single	(iii) 30% single	(iii) 23% single

Stress	100% of them reported that they went through a stressful period	100% of them reported that they went through a stressful period	100% of them reported that they went through a stressful period

Sleep	Unrefreshing sleep	Normal sleep	Normal sleep

Employment status	(i) 45% employed	(i) 51% employed	(i) 56% employed
	(ii) 55% unemployed	(ii) 49% unemployed	(ii) 44% unemployed

Obesity	None	None	None

**Table 3:** The variation in genotype frequency of *COMT* Val158Met with disease status.

Genotype	Disease status			
	FM (*N* = 20)	RH (*N* = 10)	Healthy (*N* = 10)	Fisher exact test
HH (Val/Val)	0% (*N* = 0)	50% (*N* = 5)	0% (*N* = 0)	*pvalue* = 0.033
HL (Val/Met)	65% (*N* = 13)	50% (*N* = 5)	100% (*N* = 10)	
LL (Meth/Met)	35% (*N* = 7)	0% (*N* = 0)	0% (*N* = 0)	

HH represents the genotype of normal homozygous (Val/Val); HL represents the heterozygous (Val/Met); LL represents the mutant homozygous (Meth/Met); FM: fibromyalgia patients; RH: rheumatoid patients.

## Data Availability

All data supporting the results of this article are included within this article, and any additional information is available from the corresponding authors and stored at the Department of Zoology, Faculty of Science, and University of Khartoum.

## References

[1] Liptan G. L. (2010). Fascia: a missing link in our understanding of the pathology of fibromyalgia. *Journal of Bodywork and Movement Therapies*.

[2] Hawkins R. A. (2013). Fibromyalgia: a clinical update. *Journal of Osteopathic Medicine*.

[3] Dolan L., Tung L., Raizada S. (2016). Fibromyalgia in the context of rheumatoid arthritis: a review. *Fibrom Open Access*.

[4] Di Franco M., Iannuccelli C., Bazzichi L. (2011). Misdiagnosis in fibromyalgia: a multicentre study. *Clinical & Experimental Rheumatology*.

[5] Wolfe F., Walitt B., Rasker J. J., Häuser W. (2019). Primary and secondary fibromyalgia are the same: the universality of polysymptomatic distress. *Journal of Rheumatology*.

[6] Ablin J. N., Cohen H., Buskila D. (2006). Mechanisms of disease: genetics of fibromyalgia. *Nature Clinical Practice Rheumatology*.

[7] Docampo E., Escaramís G., Gratacòs M. (2014). Genome-wide analysis of single nucleotide polymorphisms and copy number variants in fibromyalgia suggest a role for the central nervous system. *PAIN®*.

[8] Gürsoy S., Erdal E., Herken H., Madenci E., Alaşehirli B., Erdal N. (2003). Significance of catechol-O-methyltransferase gene polymorphism in fibromyalgia syndrome. *Rheumatology International*.

[9] Torpy D. J., Papanicolaou D. A., Lotsikas A. J., Wilder R. L., Chrousos G. P., Pillemer S. R. (2000). Responses of the sympathetic nervous system and the hypothalamic–pituitary–adrenal axis to interleukin‐6: a pilot study in fibromyalgia. *Arthritis & Rheumatism*.

[10] Chen J., Lipska B. K., Halim N. (2004). Functional analysis of genetic variation in catechol-O-methyltransferase (*COMT*): effects on mRNA, protein, and enzyme activity in postmortem human brain. *The American Journal of Human Genetics*.

[11] Cohen H., Neumann L., Glazer Y., Ebstein R. P., Buskila D. (2009). The relationship between a common catechol-O-methyltransferase (*COMT*) polymorphism val158met and fibromyalgia. *Clinical & Experimental Rheumatology*.

[12] Barbosa F. R., Matsuda J. B., Mazucato M. (2012). Influence of catechol-O-methyltransferase (*COMT*) gene polymorphisms in pain sensibility of Brazilian fibromialgia patients. *Rheumatology International*.

[13] Jacobsen L. M., Schistad E. I., Storesund A. (2012). The COMT rs 4680 M et allele contributes to long‐lasting low back pain, sciatica and disability after lumbar disc herniation. *European Journal of Pain*.

[14] Smith S. B., Maixner D. W., Greenspan J. D. (2011). Potential genetic risk factors for chronic TMD: genetic associations from the OPPERA case control study. *The Journal of Pain*.

[15] van Meurs J. B., Uitterlinden A. G., Stolk L. (2009). A functional polymorphism in the catechol‐O‐methyltransferase gene is associated with osteoarthritis‐related pain. *Arthritis & Rheumatism*.

[16] Sabahelzain M. M., Hamamy H. (2014). The ethnic distribution of sickle cell disease in Sudan. *The Pan African medical journal*.

[17] Mellars P. Why did modern human populations disperse from Africa ca. 60,000 years ago? A new model.

[18] Wolfe F., Smythe H. A., Yunus M. B. (1990). The American College of Rheumatology 1990 criteria for the classification of fibromyalgia. *Arthritis & Rheumatism*.

[19] Droog S., Lakenberg N., Meulenbelt I. (1996). Isolation and storage of DNA for population studies. *Fibrinolysis*.

[20] Tükel R., Gürvit H., Öztürk N. (2013). COMT Val158Met polymorphism and executive functions in obsessive-compulsive disorder. *Journal of Neuropsychiatry and Clinical Neurosciences*.

[21] Martinez-Lavin M. (2012). Fibromyalgia: When Distress Becomes (Un) Sympathetic Pain. *Pain research and treatment*.

[22] Wolfe F., Ross K., Anderson J., Russell I. J. (1995). Aspects of fibromyalgia in the general population: sex, pain threshold, and fibromyalgia symptoms. *Journal of Rheumatology*.

[23] Schiltenwolf M., Pogatzki-Zahn E. M. (2015). Pain medicine from intercultural and gender-related perspectives. *Schmerz, Der*.

[24] Aggarwal V. R., McBeth J., Zakrzewska J. M., Lunt M., Macfarlane G. J. (2006). The epidemiology of chronic syndromes that are frequently unexplained: do they have common associated factors?. *International Journal of Epidemiology*.

[25] Hâuser W., Kûhn-Becker H., von Wilmoswky H., Settan M., Brâhler E., Petzke F. (2011). Demographic and clinical features of patients with fibromyalgia syndrome of different settings: a gender comparison. *Gender Medicine*.

[26] Penrod J. R., Bernatsky S., Adam V., Baron M., Dayan N., Dobkin P. L. (2004). Health services costs and their determinants in women with fibromyalgia. *Journal of Rheumatology*.

[27] Finan P. H., Zautra A. J., Davis M. C., Lemery-Chalfant K., Covault J., Tennen H. (2011). *COMT* moderates the relation of daily maladaptive coping and pain in fibromyalgia. *PAIN®*.

[28] Inanir A., Karakus N., Ates O. (2014). Clinical symptoms in fibromyalgia are associated to catechol-O-methyltransferase (*COMT*) gene Val158Met polymorphism. *Xenobiotica*.

[29] Lee Y. H., Choi S. J., Ji J. D., Song G. G. (2012). Candidate gene studies of fibromyalgia: a systematic review and meta-analysis. *Rheumatology International*.

[30] Lee Y. H., Kim J. H., Song G. G. (2015). Association between the *COMT* Val158Met polymorphism and fibromyalgia susceptibility and fibromyalgia impact questionnaire score: a meta-analysis. *Rheumatology International*.

[31] Nicholl B. I., Holliday K. L., Macfarlane G. J. (2010). No evidence for a role of the catechol-O-methyltransferase pain sensitivity haplotypes in chronic widespread pain. *Annals of the Rheumatic Diseases*.

[32] Zhang L., Zhu J., Chen Y., Zhao J. (2014). Meta-analysis reveals a lack of association between a common catechol-O-methyltransferase (COMT) polymorphism val158met and fibromyalgia. *International Journal of Clinical and Experimental Pathology*.

[33] Vargas-Alarcón G., Fragoso J. M., Cruz-Robles D. (2007). Catechol-O-methyltransferase gene haplotypes in Mexican and Spanish patients with fibromyalgia. *Arthritis Research and Therapy*.

[34] Stein D. J., Newman T. K., Savitz J., Ramesar R. (2006). Warriors versus worriers: the role of *COMT* gene variants. *CNS Spectrums*.

[35] Daoud K. F., Barkhuizen A. (2002). Rheumatic mimics and selected triggers of fibromyalgia. *Current Pain and Headache Reports*.

[36] Fernández-de-Las-Peñas C., Ambite-Quesada S., Palacios-Ceña M. (2019). Catechol-O-methyltransferase (*COMT*) rs4680 Val158Met Polymorphism is associated with widespread pressure pain sensitivity and depression in women with chronic, but not episodic, tension-type headache. *The Clinical Journal of Pain*.

[37] Buskila D., Sarzi-Puttini P., Ablin J. N. (2006). The Genetics of Fibromyalgia Syndrome. *Future Medicine*.

[38] Xie T., Ho S. L., Ramsden D. (1999). Characterization and implications of estrogenic down-regulation of human catechol-O-methyltransferase gene transcription. *Molecular Pharmacology*.

